# Plasma Thymidine Kinase Activity as a Novel Biomarker in Metastatic Melanoma Patients Treated with Immune Checkpoint Inhibitors

**DOI:** 10.3390/cancers14030702

**Published:** 2022-01-29

**Authors:** Fernanda Costa Svedman, Marie Jalsenius, Veronica Höiom, Vitali Grozman, Mattias Bergqvist, Fabian Söderdahl, Hanna Eriksson, Samuel Rotstein, Lars Ny, Paolo A. Ascierto, Suzanne Egyhazi Brage, Hildur Helgadottir

**Affiliations:** 1 Department of Oncology and Pathology, Karolinska Institutet, 171-77 Stockholm, Sweden; fernanda.costa-svedman@regionstockholm.se (F.C.S.); veronica.hoiom@ki.se (V.H.); hanna.eriksson@regionstockholm.se (H.E.); Suzanne.Egyhazi.Brage@ki.se (S.E.B.); 2 Theme Cancer, Karolinska University Hospital, 171-76 Stockholm, Sweden; marie.jalsenius@regionstockholm.se (M.J.); samuel.rotstein@regionstockholm.se (S.R.); 3 Section of Thoracic Radiology, Department of Imaging and Physiology, Karolinska University Hospital, 171-76 Stockholm, Sweden; vitali.grozman@regionstockholm.se; 4 Department of Molecular Medicine and Surgery, Karolinska Institutet, 171-77 Stockholm, Sweden; 5 Biovica International AB, Uppsala Science Park, 752-37 Uppsala, Sweden; mattias.bergqvist@biovica.com; 6 Statisticon AB, 753-22 Uppsala, Sweden; Fabian.Soderdahl@statisticon.se; 7 Department of Oncology, Institute of Clinical Sciences, Sahlgrenska Academy at University of Gothenburg, Sahlgrenska University Hospital, 418-77 Gothenburg, Sweden; lars.ny@oncology.gu.se; 8 Department of Melanoma, Cancer Immunotherapy and Development Therapeutics, Istituto Nazionale Tumori IRCCS Fondazione “G. Pascale”, 80131 Napoli, Italy; paolo.ascierto@gmail.com

**Keywords:** melanoma, immune checkpoint inhibitors, PD-1 inhibitor, CTLA-4 inhibitor, thymidine kinase, nivolumab, pembrolizumab, ipilimumab

## Abstract

**Simple Summary:**

Immune checkpoint inhibitors (ICI) are effective in fractions of patients with disseminated melanoma. Significant toxicity can also occur from the treatments, that, in addition, are expensive. It is therefore important to increase the knowledge of predictive factors and their efficacy in different patient groups. This study is the first to analyze the plasma activity of thymidine kinase (TK), an enzyme involved in DNA synthesis and repair, as a biomarker in melanoma patients. In this study, high TK activity (TKa) levels in melanoma patients were associated with poor baseline factors, such as poor performance status, high plasma lactate dehydrogenase levels, and advanced tumor stage. High TKa levels were also associated with a poor efficacy of immune checkpoint inhibitors. TKa is hence a novel and interesting plasma biomarker in melanoma and should be further studied to define its role as a prognostic and predictive marker in this disease.

**Abstract:**

**Background.** Immune checkpoint inhibitors (ICI) are effective in fractions of patients with disseminated melanoma. This study is the first to analyze the plasma activity of thymidine kinase (TK), an enzyme involved in DNA synthesis and repair, as a biomarker in melanoma patients. **Methods.** Plasma samples were collected prior to treatment start in patients with unresectable metastatic cutaneous melanoma, treated with ICI (anti-CTLA-4 and/or anti-PD-1). Plasma TK activity (TKa) levels were determined using the DiviTum TKa ELISA assay. TKa levels were correlated with patients’ baseline characteristics, response rate (RR), progression-free survival (PFS), and overall survival (OS). **Results.** In the 90 study patients, the median TKa level was 42 Du/L (range <20–1787 Du/L). A significantly higher plasma TKa was found in patients with ECOG performance status ≥1 (*p* = 0.003), M1c-d disease (*p* = 0.015), and elevated lactate dehydrogenase levels (*p* < 0.001). The RR was 63.2% and 30.3% in those with low or high TKa, respectively (*p* = 0.022). The median PFS was 19.9 and 12.6 months in patients with low or high TKa, respectively (hazard ratio (HR) 1.83 (95% CI, 1.08–3.08), *p* = 0.024). The median OS was >60 months and 18.5 months in patients with low or high TKa, respectively (HR: 2.25 (95% CI, 1.25–4.05), *p* = 0.011. **Conclusions.** High pretreatment plasma TKa levels were significantly associated with worse baseline characteristics and poor response and survival in ICI-treated melanoma patients. TKa is hence a novel and interesting plasma biomarker in melanoma and should be further studied to define its role as a prognostic and predictive marker in this disease.

## 1. Introduction

In recent years, effective immune checkpoint inhibitor (ICI) regimens with CTLA-4 and PD-1 blocking antibodies have emerged for the treatment of melanoma [1,2,3,4,5,6]. These treatments have revolutionized the melanoma oncology field, but unfortunately a considerable fraction of melanoma patients does not respond or get lasting effects from these treatments. Significant toxicity can also occur from the treatments, that, in addition, are expensive. It is therefore important to increase the knowledge of predictive factors and their efficacy in different patient groups.

Thymidine kinase 1 (TK) is a cytosolic enzyme, a phosphotransferase that plays a pivotal role in DNA synthesis and repair [7]. TK has a key function in DNA synthesis and cell division as it is part of the reaction chain to introduce thymidine into the DNA strand [7]. Dividing cells release TK during mitotic exit, and TK can thus be detected in the blood. Further, elevated TK enzyme activity has been measured in blood samples from cancer patients and is associated with tumor proliferation and tumor burden [8]. Circulating levels of TKa, measured with the DiviTum assay, have been shown to be associated with disease stage, prognosis, and treatment efficacy in several cancer types, including breast, lung, pancreatic, and renal cell cancer [9,10,11,12,13,14,15]. This study is the first to analyze TKa levels in the plasma of melanoma patients. TKa levels in metastatic melanoma patients were measured before starting ICI treatment and correlated with baseline clinical characteristics, treatment response, and survival.

## 2. Materials and Methods

### 2.1. Patients and Plasma Samples

Plasma samples were collected from patients with unresectable metastatic cutaneous melanoma, treated with ICI (anti-CTLA-4 and/or anti-PD-1) at the Department of Oncology, Karolinska University Hospital, Stockholm, Sweden in the years 2012–2019. The treatments were administrated according to standard ICI regimens and dosage approved for the treatment of metastatic melanoma. Blood samples were taken from the patients within 5 days prior to treatment start. The blood samples were collected in EDTA tubes and centrifuged at 1500× *g* for 10 min, and the separated plasma was centrifuged at 2400× *g* for 15 min and frozen at −70 °C within 1 h of processing. The baseline clinical data included age at treatment start, gender, Eastern Cooperative Oncology Group (ECOG) performance status, baseline tumor stage according to the American Joint Committee (AJCC) on Cancer, Eighth Edition [16], number of affected organs, baseline lactate dehydrogenase (LDH) levels, previous lines of treatment, and ICI regime received after TKa sampling. The study was conducted in accordance with Good Clinical Practice, with informed consent from all patients, and was approved by the Stockholm Regional Ethics Committee.

### 2.2. TK Activity Level Analysis

Plasma TKa levels were determined using the DiviTum TKa assay (Biovica, Sweden) in accordance with the manufacturer’s instructions, which have previously been reported [7]. DiviTum TKa is a refined ELISA-based test reflecting cell proliferation rate by measuring TKa in serum, plasma, or cells. In summary, plasma was mixed with the reaction mixture in a 96-well ELISA plate, and bromodeoxyuridine (BrdU) monophosphate was generated by TK reaction, phosphorylated to BrdU triphosphate, and incorporated into a synthetic DNA strand. An anti-BrdU monoclonal antibody conjugated to the enzyme alkaline phosphatase and a chromogenic substrate were used to detect BrdU incorporation. The absorbance readings were converted using standards with known TKa values (working range from 20 to 4000 Du/L). The lower limit of detection of the assay was set at 20 Du/L, and all values below the threshold were reported as <20 Du/L. All plasma TKa analyses were performed in the Biovica laboratory (Uppsala, Sweden) where the personnel were blinded to patient and tumor data. The samples were measured in duplicate and fulfilled the Coefficient of Variation (CV) criteria of the DiviTum assay (CV < 20%). The DiviTum assay for measuring TKa is a CE-IVD-labelled assay and has also been submitted to the FDA in a 510 (k) application that is awaiting approval. Further details regarding the assay can be found at biovica.com.

### 2.3. Follow-Up

Routine follow-up after initiating the ICI treatment included monthly clinical assessments and radiological evaluations every third month. The patients had a minimum follow-up of 24 months. The patients were grouped based on TKa levels in plasma at baseline (low or high) and followed for treatment response, progression-free survival (PFS), and overall survival (OS). Best response to treatment was based on radiological investigations (CT, MRI, and/or positron emission (PET) CT tomography) evaluated by a radiologist and assessed according to the Response Evaluation Criteria in Solid Tumors (RECIST) 1.1 criteria [17]. Response rate (RR) was defined as the frequency of patients with partial (PR) or complete responses (CR) as the best response. Disease control rate (DCR) was defined as the frequency of patients with PR, CR, or stable disease (SD) as the best response after at least three months of treatment. PFS was defined as the time from treatment start until the date of confirmed progression or the date of death or of the last follow-up. OS was defined as the time from treatment start until the date of death or last follow-up.

### 2.4. Statistical Methods

A receiver operating characteristic (ROC) analysis was performed to investigate TKa cut-offs with the most optimal sensitivity and specificity to predict tumor stage, performance stage, response, and survival. Baseline characteristics and treatment responses were compared with the Chi-square test for categorical variables and the Student’s t-test for continuous variables. *p* values <0.05 were deemed statistically significant. The time to event outcomes for PFS and OS were analyzed with Kaplan–Meier curves and Cox proportional hazards regression. Median PFS and OS with 95% confidence intervals (CI) were assessed. Univariable, bivariable, and multivariable models for Cox regression were used to assess each predictor’s association with PFS and OS. Hazard ratios (HR) and corresponding two-sided 95% CI were estimated. Statistical analyses were performed with R Version 4.1.1. Concordance is a measure of the model’s predicative accuracy measured as the proportion of all evaluable pairs of subjects where the model correctly predicts a higher risk for the individual in the pair with the worst outcome.

## 3. Results

### 3.1. Baseline Characteristics

A total of 90 patients with metastatic melanoma were included in the study. The median pre-treatment plasma TKa level was 42 Du/L (range <20–1787 Du/L). There were no significant differences in TKa levels related to the age or the sex of the patients (Table 1). However, a significantly higher plasma TKa was found in patients with ECOG performance status ≥1 vs. 0–1 (*p* = 0.003), with M1c-M1d vs. M1a-M1b disease (*p* = 0.015), or with elevated vs. non-elevated LDH (*p* < 0.001). The TKa levels were higher in patients who were previously treated or had more than three affected organs, but here the difference in TKa was not significantly different. In patients with M1b-d disease, the TKa level was compared depending on if the patients had metastatic spread to a certain organ or not. This analysis was done separately from that on M1a patients in an attempt to address if the TKa level was affected by the spread to a specific organ, rather than assessing the tumor burden (as M1a patients typically have substantially less tumor burden). To conclude, no significant differences were observed regarding which organ was affected.

### 3.2. Determining the Cut-Off for TKa

**Figure 1:**
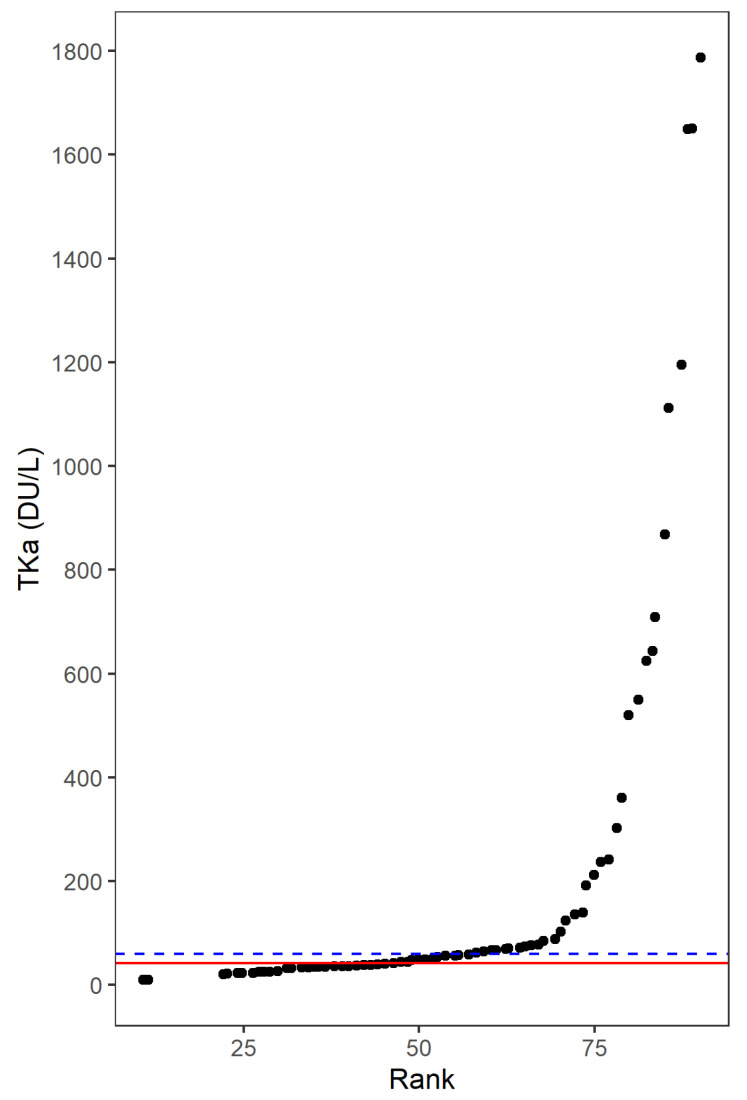
Determining the cut-off for thymidine kinase 1 activity (TKa) in the plasma of metastatic melanoma patients treated with immune checkpoint inhibitors. The figures show the rank (order) of TKa in Divitum^®^ units per liter (Du/L) on the *x*-axis and the TK value on the *y*-axis. When determining the cut-off value for TKa, the most optimal cut-off was considered as the value closest to the median that ensured a differentiation in the TKa value with sufficient numbers of observations in each group. The median TKa at baseline was 42 Du/L. This median (red line) gave a cut-off that divided patients with very similar TKa values into different groups. A cut-off of 60 Du/L (blue line) ensured more differentiation in TKa as well as enough observations in both groups.

As TKa has not been studied in melanoma before, an essential aim was to determine a suitable cut-off and then compare patients with high or low plasma TKa levels. The median TKa at baseline (42 Du/L) gave a cut-off that divided patients with very similar TKa levels into different groups (Figure 1). In that sense, 60 Du/L was considered as a more suitable cut-off, since it ensured more differentiation in TKa levels and gave sufficient numbers of patients with high or low TKa levels. In a next step, ROC analyses were performed to determine TKa cut-offs with the most optimal sensitivity and specificity in predicting baseline characteristics and outcomes (Supplementary Figure S1). The ROC analyses demonstrated that TKa cut-offs between 49 and 72 achieved the highest sensitivity and specificity to predict tumor stage, performance stage, ICI response, PFS and OS at 24 months (the median of these cut-offs was 58). The ROC analyses hence further supported the use of 60 Du/L as a reasonable cut-off.

### 3.3. Characteristics and Outcomes of Patients with High or Low TKa

In melanoma patients with high (≥60 Du/L) or low plasma TKa levels, there were no significant differences in the age, sex, or the tumor *BRAF* mutation status (Table 2). However, a high TKa level was significantly associated with ECOG performance status ≥1 (*p* < 0.001), M1c or M1d disease (*p* = 0.002), ≥3 affected organs (*p* = 0.031), elevated LDH (*p* < 0.001), and a higher median LDH (*p* < 0.001). In patients with high or low TKa, no significant differences were seen with respect to whether previous treatment lines had been received or to the ICI regime chosen for the patient. The majority of the patients were treated in first line with PD-1 inhibitor monotherapy (nivolumab or pembrolizumab). A smaller portion received a single CTLA-4 inhibitor (ipilimumab) or a CTLA-4 and PD-1 inhibitor combination (ipilimumab and nivolumab). Although there was not a statistically significant difference, more patients in the TKa-low group received combination immunotherapy (n = 6) compared to the TKa-high group (n = 0). A plausible explanation is that the better performance status and somewhat younger age of the TKa-low patients resulted in the fact that they more often were assessed and found to be able to tolerate the more toxic combination therapy.

The RR was significantly higher for patients with low TKa (63.2%) than for those with high TKa (30.3%) (*p* = 0.022) (Table 3). The rate of complete response was also higher for the TKa-low group (33.3%) than for the TKa-high group (6.0%) (*p* = 0.016). The DCR was also higher for patients with low (80.7%) vs. those with high (54.3%) TKa (*p* = 0.022). No difference was seen related to why the treatment was ended (progressive disease, adequate response, or toxicity) (Table 3).

**Figure 2:**
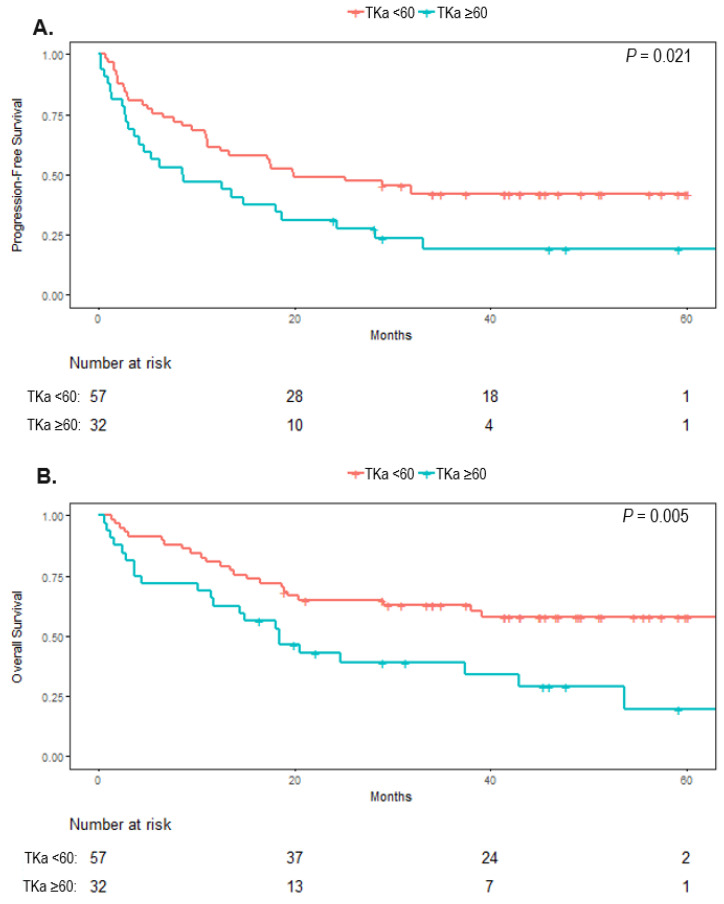
Kaplan–Meier curves for survival in melanoma patients with high (>60 Du/L) or low thymidine kinase 1 activity (TKa) in plasma before starting a treatment with immune checkpoint inhibitors. (**A**). The median progression-free survival was, in patients with low TKa, 19.9 months (95% CI, 11.0 to not reached) and in patients with high TKa, 12.6 months (95% CI, 3.6 to 28.3) (*p* = 0.021). (**B**). The median overall survival was not reached in patients with low TKa (>60 months, 95% CI, 38.0 to not reached), and in the patients with high TKa it was 18.5 (*p* = 0.005).

The median PFS was 19.9 months (95% CI, 11.0 to not reached) in patients with low TKa and 12.6 months (95% CI, 3.6 to 28.3) in patients with high TKa (*p* = 0.021) (Figure 2). The median OS was not reached (>60 months, 95% CI, 38.0 to not reached) in patients with low TKa and was 18.5 months (95% CI, 11.7 to not reached) in patients with high TKa, (*p* = 0.005).

**Figure 3:**
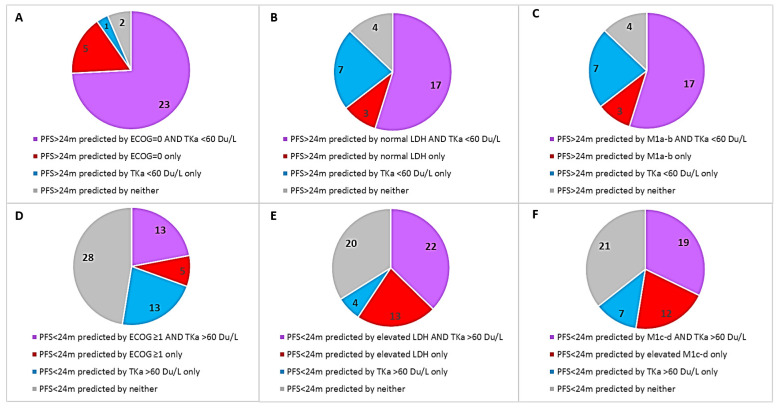
Pie charts that show the number of patients with longer (>24 months, (**A**–**C**)) or shorter (<24 months, (**D**–**F**)) progression-free survival (PFS). Each pie chart shows if longer or shorter PFS was correctly predicted by the TKa level together with one other baseline variable (ECOG, LDH, or M stage.) For example (**B**) shows that in 17 patients, long PFS was predicted by both low TKa and normal LDH at baseline (correctly predicted by both variables, purple), in 3 patients long PFS was predicted only by normal LDH, as TKa was elevated (correctly predicted only by LDH, red), in 7 patients long PFS was predicated only by low TKa, as LDH level was elevated (correctly predicted only by TKa, blue), and in 4 patients long PFS was predicted by neither variable, as both LDH and TKa levels were high (correctly predicted by none, gray).

The univariate Cox regression analysis showed significantly worse PFS and OS for patients with baseline ECOG performance status ≥1, M1c or M1d disease, elevated LDH, and a high TKa (Table 4). For TKa the HR for PFS was 1.83 (95% CI, 1.08–3.08), *p* = 0.024), and that for OS was 2.25 (95% CI, 1.25–4.05), *p* = 0.007. In the multivariate analysis, TKa was not significant for PFS or OS. A high degree of multicollinearity amongst the analyzed variables was identified as a factor that resulted in that the HR for many of the variables that were significant in the univariate model, were not significant in the multivariate model. To evaluate how the TKa was affected by each of the covariates, a bivariate regression analysis was performed where TKa was analyzed pairwise with one other baseline factor (Supplementary Figure S2). As for the PFS, TKa was only independent of the patients’ sex. Further, in the bivariable analysis for OS, TKA was independent of age, sex, and tumor stage. Figure 3 shows the number of patients with longer (>24 months, Figure 3A–C) or shorter (<24 months, Figure 3D–F) PFS; each chart shows if longer or shorter PFS was correctly predicted by the TKa level together with one other baseline variable (ECOG, LDH, or M stage). The charts show that, although there was a substantial overlap of TKa and the other variables (purple in the charts), in a fraction of patients, low or high TKa levels alone were associated with long or short PFS, respectively (blue in the charts).

## 4. Discussion

In the patients with advanced cutaneous melanoma included in the study, significantly higher TKa levels were seen in patients that at treatment start had poor performance status, more advanced tumor stage, and higher LDH level. The median TKa was 42 Du/L (range <20–1787 Du/L), while, as a reference, in 123 healthy subjects, the median TKa value was <20 Du/L (Biovica data on file). In a cohort of preoperative pancreatic cancer patients, the median TKa value was 40 Du/L, and in a cohort of preoperative renal cell cancer patients, the median TKa was 38 Du/L [12,14]. Further, in a cohort of non-small cell lung cancer patients, the median TKa was 129 Du/L before the start of systemic treatment, whereas in a cohort of breast cancer patients, the pre-treatment TKa was 57 Du/L in patients with locoregional disease and 101 Du/L in patients with visceral metastasis [15,18]. Collectively, our data show that TKa levels in patients with metastatic melanoma is, as for the other studied cancer types, elevated compared to the levels in healthy individuals and higher in patients with more advanced disease.

The patients with high TKa had a significantly poorer response to the ICI treatment and also a significantly shorter survival (both PFS and OS). In the multivariate analysis, TKa was not an independent predictor for PFS and OS. The bivariate analysis showed that TKa association with PFS and OS was, in a varying degree, dependent on ECOG, LDH, and tumor stage; however, a considerable fraction of patients did not have corresponding pairs of good or poor baseline variables (Figure 3). Clinical factors such as performance status, tumor stage, and tumor burden are well-known prognostic factors and are also predictive of ICI efficacy in melanoma [1,2,3,4,16]. As for serum markers, elevated LDH level is the strongest known prognostic and predictive factor and is the only biomarker that is routinely used when monitoring melanoma patients in the clinic and included as a marker in clinical trials [16]. The serum LDH level reflects the hypoxic environment often present in melanoma tumors, with reduced oxidative phosphorylation and increased anaerobic glycolysis, where LDH catalyzes the conversion of pyruvate to lactate when oxygen supply is low or absent [19]. LDH is not a secreted enzyme; thus, an elevated serum level is thought to be secondary to spillage of LDH when melanoma cells outgrow their blood supply. LDH is also often elevated in various conditions affecting the liver, both malignant and non-malignant [19]. The TK enzyme has a significant role in DNA synthesis and repair. These processes are highly active in proliferating cells, and dividing tumor cells release TK during mitotic exit [7,8]. Hence, LDH and TKa are both markers of cell proliferation and tumor burden, but through different cellular processes.

Several other markers have been reported as predicative for ICI efficacy, including the composition of peripheral blood leukocytes, circulating tumor DNA (ctDNA) and exosomes, tumor mutational burden (TMB), high interferon-gamma-related gene expression signature in tumors, as well as the diversity of the gut microbiome [20,21,22,23,24,25,26,27]. In the clinical setting, widespread implementation of predictive assays, such as TMB, ctDNA, exosomes, tumor RNA expression signatures, or microbiome analyses, is a challenge, e.g., due to the complex and costly techniques and equipment needed, and there are also many different assays that can be used. To compare TKa is simpler and less costly than an ELISA-based test for a single plasma marker, and the assay can readily be set up in regular hospital laboratories.

The current study is limited by a moderate sample size, and the associations found need to be verified and further defined in larger separate cohorts. In particular, it needs to be further assessed if TKa has any capacity to predict outcomes by itself or if it is chiefly associated with established clinical prognostic parameters. The cut-off levels in TKa for optimal sensitivity and specificity to predict outcomes need to be further established and verified. In this study, we only analyzed TKa before treatment start, and further analyses of the effects of TKa dynamics after treatment start will be valuable. We have initiated a study to investigate this. Additionally, this is the first study of TKa in ICI-treated patients; TKa should also be studied in patients with other types of cancers receiving immunotherapy.

## 5. Conclusions

High pretreatment plasma TKa levels were significantly associated with worse baseline characteristics and poor response and survival in ICI-treated melanoma patients. We believe that plasma TKa is an interesting, previously not explored, candidate biomarker in melanoma. Further studies are warranted to define its role as a prognostic and predictive marker in melanoma patients.

## Figures and Tables

**Table 1:** Pre-treatment thymidine kinase 1 (TK) activity (Du/L) in the plasma of patients with unresectable melanoma.

Characteristics		TK Activity (Du/L)	*p* Value
		Median (Range)	
Sex			
	Male (n = 60)	37 (<20–1787)	
	Female (n = 30)	53 (<20–869)	0.689
Age			
	≤65 years (n = 40)	37 (<20–1111)	
	>65 years (n = 50)	55 (<20–1787)	0.111
BRAF v600 mutation in tumor			
	Yes (n = 38)	46 (<20–1649)	0.649
	No (n = 52)	40 (<20–1787)	
Performance status			
	ECOG 0 (n = 70)	35 (<20–1787)	
	ECOG ≥1 (n = 20)	138 (<20–1650)	0.003
LDH			
	Normal LDH (n = 44)	34 (<20–242)	
	Elevated LDH (n = 46)	71 (<20–1787)	<0.001
Tumor stage			
	M1a or M1b (n = 48)	35 (<20–1649)	
	M1c or M1d (n = 42)	66 (<20–1787)	0.015
Numbers of affected organs			
	1–2 affected organs (n = 58)	36 (<20–1649)	
	≥3 affected organs (n = 32)	64 (<20–1787)	0.066
Affected organs (patients in stage M1b-M1d)			
	Soft tissue		
	Yes (n = 46)	47 (<20–1787)	
	No (n = 15)	59 (<20–708)	0.653
	Lung		
	Yes (n = 42)	49 (<20–1650)	
	No (n = 19)	67 (<20–1787)	0.818
	Liver		
	Yes (n = 18)	58 (<20–1787)	
	No (n = 43)	44 (<20–1195)	0.195
	Bone		
	Yes (n = 15)	67 (<20–1111)	
	No (n = 46)	42 (<20–1787)	0.886
	Brain		
	Yes (n = 13)	77 (<20–1111)	
	No (n = 48)	51 (<20–1787)	0.280
	Other		
	Yes (n = 19)	70 (<20–1787)	
	No (n = 42)	39 (<20–1650)	0.137
Previous lines of treatment			
	0 previous lines (n = 77)	37 (<20–1787)	0.261
	≥1 previous lines (n = 13)	77 (34–1650)	

**Table 2:** Characteristics of melanoma patients with low (<60 Du/L) or high thymidine kinase 1 (TK) activity (Du/L) in plasma before starting immune checkpoint inhibitor treatment.

Characteristics	TKa Low	TKa High	*p* Value
Patients, n (%)	57 (63.3%)	33 (36.7%)	
TK (Du/L), median (range)	39 (<20–59)	140 (62–1787)	<0.001
Sex, n (%)			
Male	42 (73.7%)	17 (58.6%)	0.155
Female	15 (26.3%)	12 (41.4%)	
Age, median (range)			
Age, years	64 (31–84)	71 (34–84)	0.065
Performance status, n (%)			
ECOG 0	52 (91.2%)	17 (58.6%)	<0.001
ECOG ≥ 1	5 (8.8%)	12 (41.4%)	
BRAF mutation in tumor, n (%)			
Yes	33 (57.9%)	19 (65.5%)	0.494
No	24 (42.1%)	10 (34.5%)	
Tumor stage, n (%)			
M1a or M1b	38 (66.7%)	9 (31.0%)	0.002
M1c or M1d	19 (33.3%)	20 (69.0%)	
Affected organs, n (%)			
1–2 affected organs	41 (71.9%)	14 (48.3%)	0.031
≥3 affected organs	16 (28.1%)	15 (51.7%)	
LDH, median (range)			
LDH, µkat/L	3.6 (1.7–14.2)	4.9 (3.2–37.0)	<0.001
LDH, n (%)			
Normal LDH	36 (63.2%)	7 (24.1%)	<0.001
Elevated LDH	21 (36.8%)	22 (75.9%)	
Previous lines of treatment, n (%)			
0 previous lines	52 (91.2%)	25 (86.2%)	0.517
≥1 previous lines	5 (8.8%)	4 (13.8%)	
ICI regime *, n (%)			
CTLA-4 inhibitor single	1 (1.8%)	1 (3.4%)	0.182
PD-1 inhibitor single	50 (87.7%)	28 (96.6%)	
CTLA-4 and PD-1 inhibitors	6 (10.5%)	0 (0.0%)	

* Immune checkpoint inhibitor regime that the patient started after the baseline TKa test.

**Table 3:** Response evaluations in melanoma patients with low (<60 Du/L) or high thymidine kinase 1 (TK) activity (Du/L) in plasma before starting immune checkpoint inhibitor treatment.

Response	TKa Low	TKa High	*p* Value
Best overall response, n (%)			
Complete response (CR)	19 (33.3%)	2 (6.0%)	
Partial response (PR)	17 (29.8.1%)	10 (30.3%)	
Stable disease (SD)	10 (17.5%)	6 (18.2%)	
Progressive disease (PD)	11 (19.3%)	15 (45.5%)	
Complete response rate (CR), %	33.3%	6.0%	0.016
Response rate (CR + PR), %	63.2%	30.3%	0.022
Disease control rate (CR + PR + SD), %	80.7%	54.5%	0.022
Treatment stopped due to, %			
Progressive disease	24 (42.1%)	17 (51.5%)	0.543
Adequate response	22 (38.6%)	9 (27.3%)	
Toxicity	11 (19.2%)	7 (21.2%)	

**Table 4:** Cox regressions for survival in metastatic melanoma patients treated with immune checkpoint inhibitors.

Charachteristics	Univariate Analyses			Multivariate Analyses		
	HR	95% CI	*p* Value	HR	95% CI	*p* Value
Progression-free survival						
Age (old (>65 years) vs. young)	0.89	(0.51–1.54)	0.677	0.79	(0.44–1.43)	0.444
Sex (male vs. female)	1.71	(0.99–2.96)	0.052	1.56	(0.84–2.87)	0.157
ECOG (≥1 vs. 0)	2.62	(1.44–4.74)	0.002	2.04	(0.93–4.45)	0.075
Tumor stage (M1a-b vs. M1c-d)	2.01	(1.17–3.46)	0.012	1.63	(0.89–2.98)	0.116
LDH (elevated vs. normal)	1.88	(1.12–3.15)	0.017	1.99	(1.12–3.55)	0.019
TK activity (high (<60 Du/L) vs. low)	1.83	(1.08–3.08)	0.024	0.78	(0.39–1.56)	0.484
Overall survival						
Age (old (>65 years) vs. young)	1.22	(0.67–2.21)	0.517	1.14	(0.60–2.17)	0.688
Sex (male vs. female)	2.52	(1.31–4.85)	0.006	2.35	(1.13–4.85)	0.021
ECOG (≥1 vs. 0)	3.42	(1.77–6.60)	<0.001	2.07	(0.87–4.91)	0.099
Tumor stage (M1a-b vs. M1c-d)	2.99	(1.55–5.73)	0.001	2.22	(1.09–4.54)	0.029
LDH (elevated vs. normal)	2.29	(1.26–4.15)	0.006	2.15	(1.12–4.14)	0.021
TK activity (high (<60 Du/L) vs. low)	2.25	(1.25–4.05)	0.007	0.82	(0.39–1.75)	0.613

## Data Availability

All data relevant to the study are included in the article or uploaded as supplementary information.

## Supplementary Materials

The following supporting information can be downloaded at: https://www.mdpi.com/article/10.3390/cancers14030702/s1, Supplementary Figure S1: ROC curve analysis with the most optimal TKa cut-offs for the sensitivity and specificity in predicting performance stage, tumor stage, treatment response, progression-free survival, and overall survival. Supplementary Figure S2: Bivariable regression with pairwise analyses of TKa together with one other variable for progression-free survival and overall survival in metastatic melanoma patients treated with immune checkpoint inhibitors.
