# Supplementary material for: Plasma Thymidine Kinase Activity as a Novel Biomarker in Metastatic Melanoma Patients Treated with Immune Checkpoint Inhibitors
Source: Cancers (Basel). 2022 Jan 29;14(3):702. doi: 10.3390/cancers14030702 (PMC8833501; doi:10.3390/cancers14030702)
Supplement: Supplementary file 1 [file cancers-14-00702-s001.zip › cancers-1533935-supplementary.pdf]

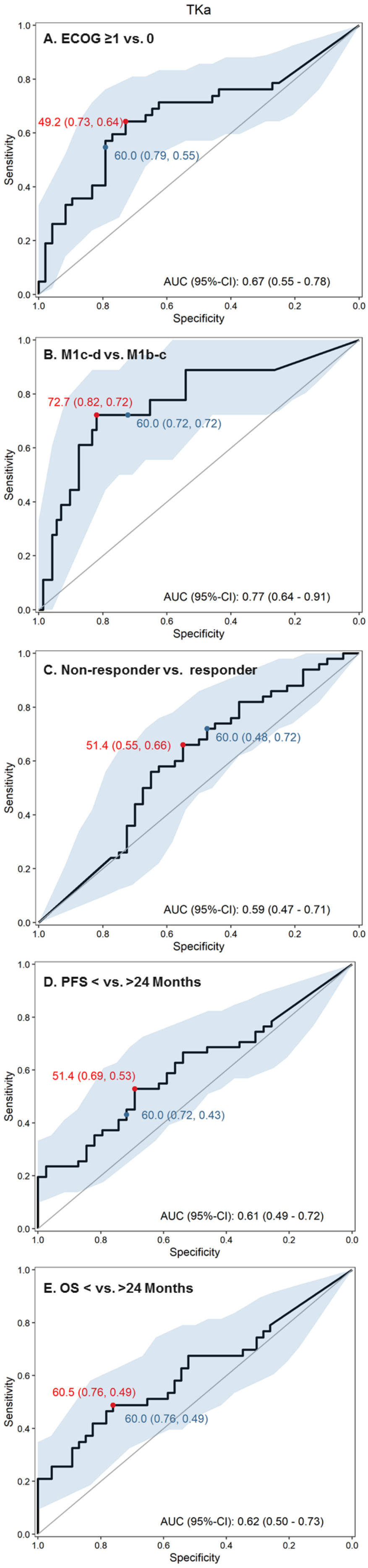
TKa
A. ECOG ≥1 vs. 0
49.2 (0.73, 0.64)
60.0 (0.79, 0.55)
AUC (95%-CI): 0.67 (0.55 - 0.78)
B. M1c-d vs. M1b-c
72.7 (0.82, 0.72)
60.0 (0.72, 0.72)
AUC (95%-CI): 0.77 (0.64 - 0.91)
C. Non-responder vs. responder
51.4 (0.55, 0.66)
60.0 (0.48, 0.72)
AUC (95%-CI): 0.59 (0.47 - 0.71)
D. PFS < vs. >24 Months
51.4 (0.69, 0.53)
60.0 (0.72, 0.43)
AUC (95%-CI): 0.61 (0.49 - 0.72)
E. OS < vs. >24 Months
60.5 (0.76, 0.49)
60.0 (0.76, 0.49)
AUC (95%-CI): 0.62 (0.50 - 0.73)
Sensitivity
Specificity

Supplementary Figure S1. ROC curve analysis with the most optimal TKa cut-offs for the sensitivity and specificity in predicting: **A.** Performance stage (ECOG ≥1 vs.ECOG 0) **B**. Tumor stage (M1c-d vs. M1a-b),) **C.** Response (SD and PD vs. CR and PR), **D.** Progression-free survival (shorter or longer than 24 months) and **E.** Overall survival (shorter or longer than 24 months). The optimal TKa cut-off for each of the analysis is shown in red font and the TKa of 60.0 that was selected as a cut-off for the comparative analyses in the study is shown in blue.

PFS

Term	HR	95%-CI	P-value
TKa (high vs. low)	1.66	(0.97 - 2.83)	0.066
Age (old vs. young)	1.53	(0.88 - 2.68)	0.134
TKa (high vs. low)	1.91	(1.12 - 3.26)	0.017
Sex (female vs. male)	0.79	(0.45 - 1.37)	0.397
TKa (high vs. low)	1.24	(0.66 - 2.34)	0.505
ECOG (≥1 vs. 0)	2.29	(1.13 - 4.65)	0.022
TKa (high vs. low)	1.57	(0.90 - 2.71)	0.109
Tumor stage (M1a-b vs. M1c-d)	1.65	(0.96 - 2.84)	0.071
TKa (high vs. low)	1.32	(0.73 - 2.37)	0.354
LDH (elevated vs. normal)	1.83	(1.03 - 3.27)	0.040

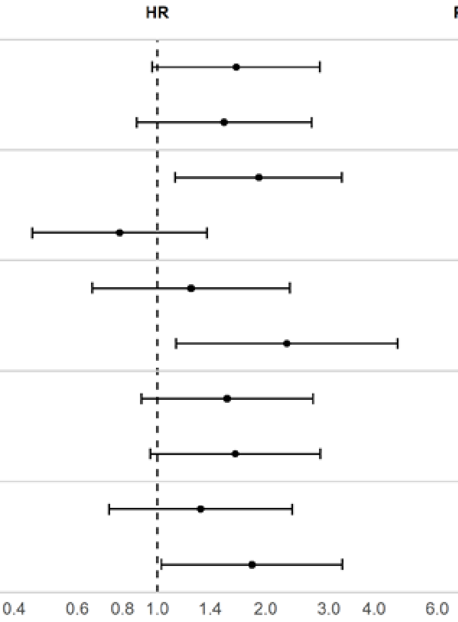

OS

Term	HR	95%-CI	P-value
TKa (high vs. low)	1.89	(1.04 - 3.46)	0.038
Age (old vs. young)	2.18	(1.12 - 4.25)	0.023
TKa (high vs. low)	2.23	(1.23 - 4.07)	0.009
Sex (female vs. male)	1.04	(0.57 - 1.92)	0.891
TKa (high vs. low)	1.41	(0.69 - 2.89)	0.346
ECOG (≥1 vs. 0)	2.76	(1.25 - 6.06)	0.012
TKa (high vs. low)	1.90	(1.04 - 3.49)	0.038
Tumor stage (M1a-b vs. M1c-d)	1.96	(1.06 - 3.63)	0.031
TKa (high vs. low)	1.49	(0.77 - 2.87)	0.234
LDH (elevated vs. normal)	2.63	(1.32 - 5.24)	0.006

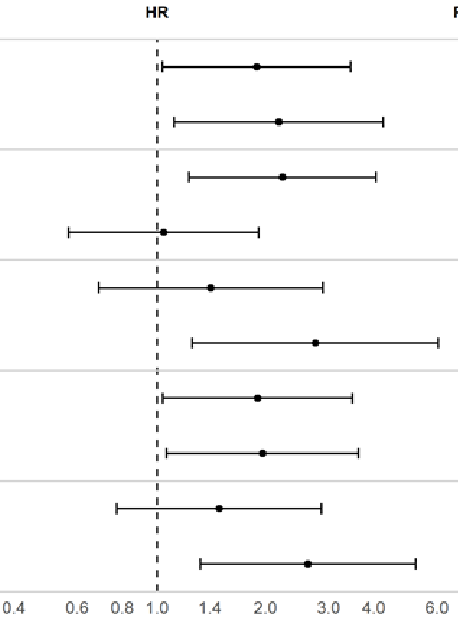

Supplementary Figure S2. Bivariable regression with pairwise analyses of TKa together with one other variable for **A.** progression-free survival (PFS) and **B.** overall survival (OS) in metastatic melanoma patients treated with immune checkpoint inhibitors.
